# Idea of biomineralization-inspired fabrication for ceramics at room or low temperature

**DOI:** 10.1093/nsr/nwaf428

**Published:** 2025-10-11

**Authors:** Zhaoyong Zou, Hang Ping, Qunfeng Cheng, Huailing Gao, Yufang Zhu, Zhengyi Fu

**Affiliations:** State Key Laboratory of Advanced Technology for Materials Synthesis and Processing, Wuhan University of Technology, China; State Key Laboratory of Advanced Technology for Materials Synthesis and Processing, Wuhan University of Technology, China; State Key Laboratory of Bioinspired Interfacial Materials Science, Suzhou Institute for Advanced Research, University of Science and Technology of China, China; School of Chemistry and Materials Science, University of Science and Technology of China, China; Institute of Energy Materials Science (IEMS), University of Shanghai for Science and Technology, China; CAS Key laboratory of Mechanical Behavior and Design of Materials, Department of Modern Mechanics, Hefei National Research Center for Physical Sciences at the Microscale, University of Science and Technology of China, China; State Key Laboratory of High Performance Ceramics, Shanghai Institute of Ceramics, Chinese Academy of Sciences, China; State Key Laboratory of Advanced Technology for Materials Synthesis and Processing, Wuhan University of Technology, China

## Abstract

This perspective outlines a visionary roadmap for making ceramics at room temperature, inspired by nature's energy-efficient strategies, to overcome the high energy costs and material limitations of traditional sintering.

Ceramic materials play an indispensable role in daily life, industrial production and advanced technologies. Traditionally, ceramic sintering is the densification of compacted powder at high temperatures, in which interparticle pores are eliminated by atomic diffusion driven by thermal energy [1]. However, this process is highly energy-intensive and contributes significantly to global CO_2_ emissions. Moreover, although temperature-induced atomic diffusion promotes densification, it also leads to uncontrolled grain growth—a fundamental trade-off in sintering dynamics [2]. In polycrystalline ceramics, high density generally enhances mechanical properties such as strength and toughness, whereas excessive grain growth deteriorates them. Therefore, developing novel fabrication routes for ceramics that operate at room or low temperature is of critical scientific and practical importance.

In contrast to traditional sintering process, living organisms, such as shells, teeth and bones, synthesize dense biominerals through biomineralization at ambient temperatures. Furthermore, these biological materials usually exhibit exquisite microstructures and an excellent combination of conflicting mechanical properties, such as hardness and toughness. For example, natural nacre consists of ∼95 wt% aragonite tablets arranged in a brick-and-mortar pattern, with 5 wt% of organic material serving as a flexible binder. This unique structure has a hardness of 2.5–4.5 GPa and a flexural strength of 80–140 MPa, which are 3000 times higher than those of sintered calcium carbonate [3]. Thus, through billions of years of evolution, biological systems have developed efficient and sophisticated strategies to tackle the main problems of traditional ceramic sintering.

***Bioinspired materials***. Over the past two decades, pioneered by Prof. Lei Jiang and Prof. Joanna Aizenberg *et al.*, numerous bioinspired materials with excellent mechanical and functional properties have been developed. The core concept of bioinspired materials is to understand the unique structure–function relationship of natural biological materials and replicate these features by using laboratory-based techniques. For example, inspired by the brick-and-mortar structure of nacre, Ritchie *et al.* fabricated nacre-like a Polymethyl Methacrylate (PMMA)–Al_2_O_3_ composite, through freeze-casting followed by sintering, that exhibited significantly higher fracture toughness compared with conventional laminated ceramics [3]. Similarly, drawing inspiration from water striders, spiders’ silk and other natural systems, Prof. Jiang’s group developed various superhydrophobic and bioinspired interfacial materials [4]. These achievements highlight the versatility of bioinspired materials in solving engineering challenges.

***Bioprocessing-inspired fabrication.*** Furthermore, besides the structure–function relationship, the structure-forming processes of biological materials at room temperature are equally fantastic. Recently, the research group of Prof. Zhengyi Fu has proposed a new research direction known as ‘bioprocessing-inspired fabrication’ [5]. The idea is to develop new material synthesis and preparation technologies by learning from biological fabrication processes or the relationship between biological fabrication processes and biostructures (Fig. 1A).

**Figure 1.**
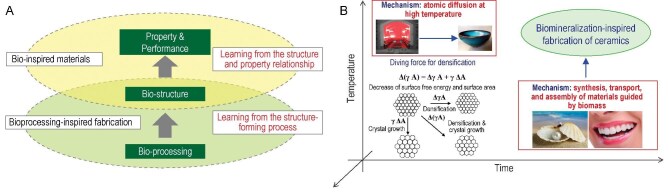
(A) Idea of bioprocessing-inspired fabrication of materials and (B) biomineralization-inspired fabrication of ceramics at room and low temperature.

***Biomineralization-inspired fabrication for ceramics.*** Under the framework of the above research direction, we further propose developing biomineralization-inspired fabrication technologies for ceramics at room or low temperature. The traditional high-temperature sintering and natural biomineralization processes have fundamental differences: the former relies on thermal energy to drive atomic diffusion, whereas the latter involves the synthesis, transport and assembly of materials guided by biomass. In biomineralization, organic molecules, such as peptides, proteins and polysaccharides, play pivotal roles in directing ion transport, controlling nucleation and growth, and orchestrating the assembly of building blocks. However, the underlying mechanisms remain complex and not fully understood. Thus, learning the key processes will inspire novel fabrication strategies for ceramics under mild conditions. This emerging research direction mainly includes the following four aspects (Fig. 1B): (i) How does natural bioprocessing work? Revealing the biomineralization strategies used by living organisms that allow the room-temperature fabrication of biominerals. (ii) What can we learn for artificial work? Learning the rules and principles of biomineralization for controlling the synthesis, transport and assembly of materials. (iii) What new methods can we develop? Novel biomineralization-inspired fabrication techniques for ceramic materials at room or low temperature. (iv) Fabrication of large-scale ceramic materials for various applications.

We aim to address three fundamental scientific issues: (i) key regulatory factors guiding the room-temperature synthesis and hierarchical organization of biominerals, including biological units, growth factors, transportation pathways and confined conditions, etc.; (ii) design principles of the building blocks of ceramics and corresponding regulation mechanisms, including the construction of organic/inorganic interfaces, ordered assembly of building blocks and stabilization mechanisms of complex structures; (iii) densification mechanism of ceramics at room temperature under artificial confined environments or external fields, including mass transport, reaction kinetics and assembly dynamics. It is evident that biomineralization-inspired ceramic fabrication represents a multidisciplinary research frontier integrating materials science and biology, which will overturn the traditional high-temperature sintering paradigm that has dominated ceramic processing for millennia, and may open new pathways for innovative and sustainable materials synthesis.

Significant progress has been made in developing bioprocessing-inspired fabrication techniques. For instance, living organisms have been used as biological platforms to direct materials synthesis, in which nitrogen-doped anatase TiO_2_ was successfully synthesized from amorphous precursors by using living mussels [5]. Inspired by molluscan nacre formation, Yu and co-workers developed a consecutive ‘assembly-and-mineralization’ approach to fabricate millimeter-thick synthetic nacre [6]. Inspired by the role of water in biomineralization, Yang *et al.* developed a nanoconfined water-induced alignment strategy to fabricate MXene-bridged graphene sheets at room temperature [7]. Inspired by the use of amorphous precursors in biomineralization, Zou *et al.* discovered a previously unknown crystalline phase—calcium carbonate hemihydrate (CCHH, CaCO_3_·½H_2_O)—highlighting the potential of amorphous precursors for synthesizing new materials [8]. Tang *et al.* further manufactured inorganic monoliths of amorphous phases at room temperature by using particle fusion while regulating the structurally bound water and external pressure [9]. They also constructed continuously structured inorganic materials by cross-linking ionic oligomers [10]. Fascinated by the hierarchical columnar structure of tooth enamel, Yeom *et al.* performed *ex vivo* replication of enamel-inspired columnar nanocomposites by using the sequential growth of zinc oxide nanowire carpets followed by the layer-by-layer deposition of a polymeric matrix [11]. Furthermore, learning from the fact that the hydroxyapatite nanowires in natural enamel are interconnected by an amorphous intergranular phase, Zhao *et al.* engineered artificial tooth enamel through the assembly of amorphous intergranular phase-coated hydroxyapatite nanowires intertwined with polyvinyl alcohol [12]. Ping *et al.* investigated the mineralization of inorganic materials within collagen fibrils and revealed that intrafibrillar mineralization induces collagen contraction, generating stresses of up to several megapascals [13]. Taking this as inspiration, we further propose the idea of macroscale and microscale prestressed ceramics: to form compressive prestress in the macro‐ or microscale range in ceramics by using a designed additional force, which offsets the fracture stress at the crack tips and then enhances the strength of the ceramics [14].

***Future research perspective***. Based on the current research progress, future research should focus on the following aspects. (i) Elucidating the formation mechanisms of natural biominerals—a fundamental understanding of how biological systems form minerals with sophisticated hierarchical architectures and superior mechanical properties is essential. This requires in-depth investigation of the compositional and microstructural evolution across different growth stages, thus uncovering key regulatory factors—such as the role of specific biomolecules, ion-transport pathways and the driving forces behind densification processes. (ii) Biomineralization-inspired synthesis of inorganic materials—emphasis should be placed on the rational design and synthesis of organic/inorganic templates that mimic the functions of biological molecules. Such biomimetic agents can be used to regulate nucleation, growth and reaction kinetics, enabling precise control over the microstructure, morphology and properties of the resulting materials. (iii) Development of low-temperature synthesis, assembly and densification technologies—innovative approaches should be designed to facilitate material transport, reaction and densification under mild conditions, such as constructing confined micro-/nanoscale reaction environments and employing external fields (e.g. light, mechanical force, electric field). (iv) Equipment design and process optimization for macroscopic ceramic fabrication—there is a need to advance the design of specialized equipment capable of producing large-scale ceramic components at room or low temperatures. Promising applications encompass biomedical devices, energy-efficient construction materials and underwater protective coatings, where room-temperature processing offers critical advantages in efficiency, functionality and compatibility with heat-sensitive components. Finally, advancing this field will require close collaboration between biologists, chemists and materials scientists to translate biomineralization principles into practical fabrication technologies.

## References

[1] Kingery WD . J Appl Phys 1959; 30: 301–6. 10.1063/1.1735155

[2] Ji W, Rehman SS, Wang W et al. Sci Rep 2015; 5: 15827. 10.1038/srep15827 26503706 PMC4622079

[3] Munch E, Launey ME, Alsem DH et al. Science 2008; 322: 1516–20. 10.1126/science.1164865 19056979

[4] Gao X, Jiang L. Nature 2004; 432: 36. 10.1038/432036a 15525973

[5] Xie J, Ping H, Tan T et al. Prog Mater Sci 2019; 105: 100571. 10.1016/j.pmatsci.2019.05.004

[6] Mao L, Gao H, Yao H et al. Science 2016; 354: 107–10. 10.1126/science.aaf8991 27540008

[7] Yang J, Li M, Fang S et al. Science 2024; 383: 771–7. 10.1126/science.adj3549 38359121

[8] Zou Z, Habraken WJEM, Matveeva G et al. Science 2019; 363: 396–400. 10.1126/science.aav0210 30679371

[9] Mu Z, Kong K, Jiang K et al. Science 2021; 372: 1466–70. 10.1126/science.abg1915

[10] Liu Z, Shao C, Jin B et al. Nature 2019; 574: 394–8. 10.1038/s41586-019-1645-x 31619792

[11] Yeom B, Sain T, Lacevic N et al. Nature 2017; 543: 95–8. 10.1038/nature21410 28252079

[12] Zhao H, Liu S, Wei Y et al. Science 2022; 375: 551–6. 10.1126/science.abj3343 35113708

[13] Ping H, Wagermaier W, Horbelt N et al. Science 2022; 376: 188–92. 10.1126/science.abm2664 35389802

[14] Gu J, Fu S, Ping H et al. Interdiscip Mater 2024; 3: 897–906. 10.1002/idm2.12224

